# Effects of Docetaxel Combined with Icotinib on Serum Tumor Markers and Quality of Life of Patients with Advanced Non-Small Cell Lung Cancer

**DOI:** 10.18502/ijph.v49i10.4691

**Published:** 2020-10

**Authors:** Huawei LIN, Jing CHANG, Jun LI

**Affiliations:** 1. Department of Medicine, Shandong Medical College, Jinan 250002, P.R. China; 2. Department of Medical Oncology, The Third Affiliated Hospital, Shandong First Medical University (Affiliated Hospital of Shandong Academy of Medical Sciences), Jinan 250031, P.R. China

**Keywords:** Docetaxel, Icotinib, Paclitaxel, Carboplatin, Cancer

## Abstract

**Background:**

To investigate the effects of docetaxel combined with icotinib on tumor markers in serum and quality of life of patients with advanced non-small cell lung cancer (NSCLC).

**Methods:**

Overall, 121 patients with advanced NSCLC, admitted to the Third Affiliated Hospital of Shandong First Medical University, China from 2017–2018 were selected as subjects. Among them, 58 patients treated with docetaxel combined with icotinib for chemotherapy were considered as study group, and 63 patients treated with paclitaxel combined with carboplatin as control group. The clinical efficacy, adverse reactions, and ECOG scores of the two groups were observed. CEA, CA125, and SCC (Tumor markers) levels of the two groups before and after treatment were detected by chemiluminescence immunoassay (CLIA).

**Results:**

The leukopenia, oral mucosa ulcer and mild numbness in the control group were significantly higher than those in the study group (*P*<0.05). After treatment, ECOG scores of both groups decreased (*P*<0.05), and the ECOG score of the study group was significantly higher than that of the control group (*P*<0.05). The serum CEA, CA125 and SCC levels of the study group and the control group after treatment decreased significantly compared with that before treatment (*P*<0.05).

**Conclusion:**

Application of docetaxel combined with icotinib for chemotherapy of patients with advanced NSCLC can effectively reduce the serum levels of CEA, SCC, and the CA125. Docetaxel combined with icotinib can significantly reduce adverse reactions and better improve the quality of life of patients compared with paclitaxel combined with carboplatin, which is worthy of clinical promotion.

## Introduction

Lung cancer is one of the malignant tumors with increasing incidence and great harm to human health. The most common type of lung cancer is non-small cell lung cancer (NSCLC). Compared with small cell lung cancer, the growth and division rate of NSCLC cancer cells are slow and the metastasis is relatively late (1). Clinically, the most common NSCLC types are adenocarcinoma, large cell carcinoma and squamous cell carcinoma (squamous carcinoma) (2). Most of the early stage NSCLC lesions are occult and have progressed to advanced stages when diagnosed (3).

Chemotherapy is one of the commonly used ways to treat cancer (4). Paclitaxel combined with carboplatin is the main treatment for advanced NSCLC, which can effectively prolong the survival of patients and improve their quality of life. However, its toxic reaction is relatively strong, which will cause great discomfort to patients, and some patients are prone to develop chemotherapy resistance or chemotherapy intolerance due to their own factors, resulting in the failure to achieve the expected good efficacy (5). Therefore, a new treatment method is of great importance for the treatment of advanced NSCLC patients.

Docetaxel is a class of drugs approved for the treatment of advanced NSCLC with metastatic or failed primary chemotherapy, and is currently widely used in clinical practice. Docetaxel is a paclitaxel antitumor agent that achieves the desired therapeutic effect by interfering with the microtubule network necessary for cell function during mitosis (6). At present, targeted therapy has been widely used in clinical practice, and targeted drug therapy for advanced NSCLC has been widely utilized in clinical trials (7). Epidermal growth factor receptor (EGFR) is a tyrosine kinase that is usually up-regulated in non-small cell lung cancer (8). Icotinib hydrochloride, a molecular target drug for lung cancer that has attracted much attention, is a new generation of targeted anticancer drug by inhibiting EGFR kinases, and its first adaptation disease is advanced NSCLC(9).

At present, there are many clinical applications of docetaxel and icotinib, but the efficacy and safety of the combined application of them in patients with advanced NSCLC need to be further studied. In this study, docetaxel combined with icotinib were used to treat advanced NSCLC. It was compared with traditional paclitaxel combined with carboplatin to observe and compare the therapeutic effect, adverse reactions and the effect on tumor markers in serum of the two treatment schemes.

## Material and Methods

### General data

Overall121 patients with advanced NSCLC admitted to The Third Affiliated Hospital of Shandong First Medical University, China from 2017–2018 were enrolled as study subjects. Among them, 58 patients treated with docetaxel combined with icotinib for chemotherapy were considered as study group, and 63 patients treated with paclitaxel combined with carboplatin were considered as control group. There were 34 males and 29 females in the study group, aged 30–71 yr, with an average age of (46.96±7.71) yr. In the control group, there were 30 males and 28 females, aged 38–69 yr, with an average age of (47.94±8.61) yr.

### Inclusion and exclusion criteria

#### Inclusion criteria

Patients with advanced NSCLC diagnosed by cytology, histology and iconography (10). Patients with complete clinical data.

#### Exclusion criteria

Patients who were allergic to the treatment drugs. Patients with severe damage to heart, liver, brain and other organs. Patients with other malignant tumors, pulmonary abscesses, pulmonary heart disease, pulmonary tuberculosis, pneumonia, pulmonary fibrosis or severe infection. Patients with mental illness or family history of mental illness.

This study had been informed to patients and their family before being conducted, and patients themselves had signed the informed consent. This study was unanimously approved by the Ethics Committee of our hospital.

#### Therapies

Patients in the study group received docetaxel (Beijing Union Pharm, SFDA approval number: H20093738) by intravenous infusion, which was given at 65 mg/m^2^ for 1 h and repeated once every 3 weeks. Then, patients were treated with icotinib hydrochloride (Betta Pharmaceuticals Co., Ltd, H20110061), with 125 mg each time, three times per day on an empty stomach. One course of treatment was 21 d, and there were 6 courses of treatment. Patients in the control group were given intravenous carboplatin (Kunming Guiyin Pharmaceutical Co., Ltd., SFDA approval number: H20053908) 350 mg/m^2^ and taxol injection (Jiangsu Hongdoushan Pharmaceutical Co., Ltd., SFDA approval number: H20067345) 200mg/m^2^. One course of treatment was 21 d, and there were 6 courses of treatment. Docetaxel, carboplatin and paclitaxel were all diluted with saline or 5% glucose before injection. In the course of chemotherapy, the amount of dosage would be reduced the amount or stopped for observation if patients felt uncomfortable. During the course of treatment, the changes in various indicators of the patients’ body would be constantly paid attention to.

#### Efficacy evaluation and outcome measures

Efficacy of patients were evaluated after 3 months of chemotherapy, and the it was divided into complete response (CR), partial response (PR), stable disease (SD) and progression disease (PD) according to the criteria provided by the WHO (11). PD: The maximum diameters of the target lesions increased by at least 20%, new lesions appeared. SD: The maximum diameters of target between PR and PD. PR: The maximum diameters of target lesions reduced by ≥30% and maintained for at least 4 weeks. CR: All target lesions disappeared, no new lesions appeared, the tumor markers were normal and maintained for at least 4 weeks. The total effective rate was calculated by the sum of CR and PR. The total effective rate = (CR+PR)/the total number of cases ×100%.

The physical condition of the two groups of patients before and after treatment was evaluated by ECOG score, which scored 0, 1, 2, 3, 4, and 5. The lower the score was, the better the improvement of the quality of life of the patients after treatment was (12). As for the evaluation of adverse reactions in the course of chemotherapy, it mainly included nausea, vomiting, loss of appetite, abdominal pain, diarrhea, oral mucosa ulcer, pharyngitis, etc.

#### Detection index

In both groups, 5ml venous blood was extracted in the morning before and after treatment in an empty stomach. It was placed and centrifuged in a centrifuge at a speed of 3500 r/min and stored in the refrigerator. CEA was detected with Roche Cobas8000 chemiluminescence immunoassay (CLIA) and CEA kit (Shanghai Jingkang Bioengineering Co., Ltd., JK-(a)-4672). CA125 was detected with Roche Cobas CLIA and CA125 kit (Shanghai Jingkang Bioengineering Co., Ltd., IK-(a)-4558). SCC was detected with Abbott i2000 CLIA and SCC kit (Tellgen, 20163402328).

#### Statistical methods

SPSS 22.0 (IBM Corp, Armonk, NY, USA) was used for statistical analysis. Enumeration data was expressed by [n (%)]. Measurement data was expressed by mean ± standard deviation (x±sd). Enumeration data between groups were qualified by chi-square test, and enumeration data between groups were qualified by independent sample *t*-test. The paired *t-*test was used for intragroup comparison. When *P*<0.05, the difference was statistically significant.

## Results

### Baseline data of the two groups

In the study group, there was no significant difference in gender, body mass index, age, smoking history, marriage history, drinking history, place of residence, pathological pattern and TNM stage (Table 1).

**Table 1:** Comparison of clinical baseline data in the study group and the control group [n(%)]/(x̄±sd)

***Groups***	***Study group (n=63)***	***Control group (n=58)***	***t/χ^2^***	***P***
Gender			0.061	0.805
Female	34(53.97)	30(51.72)		
Male	29(46.03)	28(48.28)		
BMI (kg/m^2^)	19.96±2.01	20.42±1.28	1.487	0.140
Age (yr)	46.96±7.71	47.94±8.61	0.661	0.510
Smoking history			0.011	0.917
Yes	44(69.84)	40(68.97)		
No	19(30.16)	18(31.03)		
Marital history			0.053	0.818
Unmarried	13(20.63)	11(18.97)		
Married	50(79.37)	47(81.03)		
Drinking history			0.022	0.882
Yes	35(55.56)	33(56.90)		
No	28(44.44)	25(43.10)		
Place of residence			0.136	0.712
Cities	39(61.90)	34(58.62)		
Countrysides	24(38.10)	24(41.38)		
Pathological pattern			0.364	0.834
Large cell carcinoma	13(20.63)	10(17.24)		
Squamous cell Carcinoma	31(49.21)	28(48.28)		
Adenocarcinoma	19(30.16)	20(34.48)		
TNM stage			1.589	0.208
Grade IIIb	23(36.51)	15(25.86)		
Grade IV	40(63.49)	43(74.14)		

### Clinical efficacy of the two groups

After chemotherapy, the total number of effective patients in the study group was 45 (71.43%), while that in the control group was 36 (62.07%). There was no significant difference in the total clinical effective rate between the study group and the control group (Table 2).

**Table 2:** Comparison of clinical efficacy between the two groups [n(%)]

***Groups***	***CR***	***PR***	***SD***	***PD***	***Total effective rate***
Research group (n=63)	16(25.40)	29(46.03)	10(15.87)	8(12.70)	45(71.43)
Control group (n=58)	14(24.14)	22(37.93)	13(22.41)	9(15.52)	36(62.07)
χ^2^	-	-	-	-	1.195
*P*	-	-	-	-	0.274

### Adverse reaction rate of the two groups

During the course of chemotherapy, some patients in both groups had different adverse reactions. In the study group, there were 44 cases (69.84%) of nausea, 12 cases (19.05%) of leukocytopenia, 14 cases (22.22%) of loss of appetite, 20 cases (31.75%) of abdominal pain, 10 cases (15.87%) of diarrhea, 15 cases (23.81%) of oral mucosa ulcer, and 6 cases (9.52%) of mild numbness. In the control group, there were 41 cases (70.69%) of nausea, 20 cases (34.48%) of leukopenia, 18 cases (31.03%) of loss of appetite, 23 cases (39.66%) of abdominal pain, 9 cases (15.52%) of diarrhea, 25 cases (43.10%) of oral mucosa ulcer, and 28 cases (48.28%) of mild numbness.

The incidence of nausea, loss of appetite, abdominal pain and diarrhea in the study group was not significantly different from that in the control group, while the incidence of leukopenia, oral mucosa ulcer and mild numbness in the control group was significantly higher than that in the study group, with statistical significance (*P*<0.05) (Table 3).

**Table 3:** Comparison of the incidence of adverse reactions between the study group and the control group during treatment [n(%)]

***Class***	***Research group (n=63)***	***Control group (n=58)***	***χ^2^***	***P***
Nausea	44(69.84)	41(70.69)	0.010	0.919
Leukocytopenia	12(19.05)	20(34.48)	3.937	0.047
Anepithymia	14(22.22)	18(31.03)	1.206	0.272
Abdominal pain	20(31.75)	23(39.66)	0.825	0.364
Diarrhea	10(15.87)	9(15.52)	0.003	0.957
Oral mucosa ulcer	15(23.81)	25(43.10)	5.080	0.024
Mild numbness	6(9.52)	28(48.28)	22.451	<0.001

### Comparison of ECOG scores before and after treatment between the two groups

**Fig. 1:**
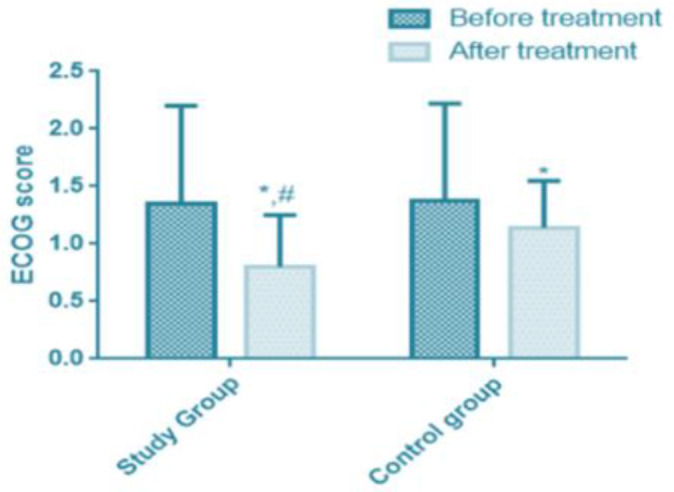
Comparison of ECOG scores before and after treatment between the study group and the control group ECOG scores of the study group and the control group decreased after treatment (*P*<0.05). The ECOG score of the study group was significantly higher than that of the control group (*P*<0.05). Note: ^*^ compared with before treatment, *P*<0.05; # compared with the control group after treatment, *P*<0.05

The ECOG score was (1.35±0.85) before treatment and (0.80±0.45) after treatment in the study group. The ECOG score of was (1.38±0.84) before treatment and (1.14±0.41) after treatment in the control group. ECOG scores of both groups decreased after treatment, with statistically significant differences (*P*<0.05). The ECOG score of the study group was significantly higher than that of the control group (*P*<0.05) (Table 4 and Fig. 1).

**Table 4:** Comparison of ECOG score before and after treatment between the two groups (x̄±sd, score)

***Groups***	***Before treatment***	***After treatment***	***t***	***P***
Research group (n=63)	1.35±0.85	0.80±0.45	4.539	<0.001
Control group (n=58)	1.38±0.84	1.14±0.41	2.038	0.044
*t*	0.195	4.332	-	-
*P*	0.846	<0.001	-	-

### Changes of tumor markers in the two groups before and after treatment

**Fig. 2:**
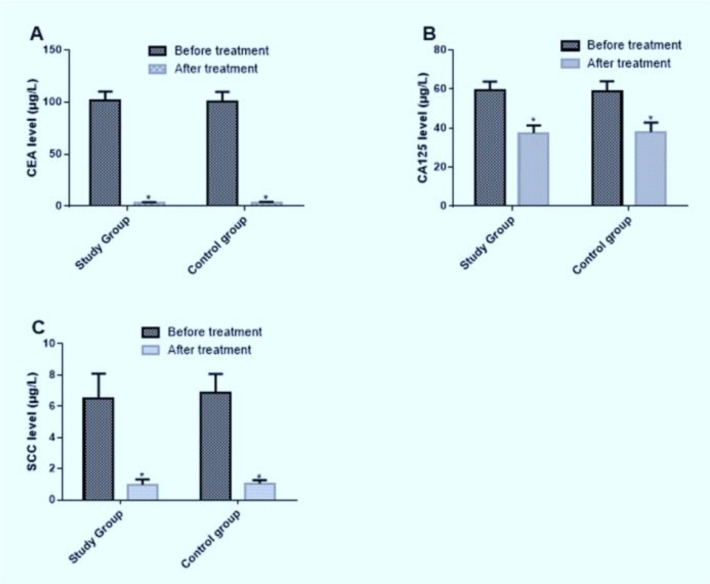
Changes of tumor markers in the study group and the control group before and after treatment A: The comparison of levels of serum CA125 after treatment between the study group and the control group. The serum CA125 levels of the study group and the control group after treatment decreased significantly compared with those before treatment (*P*<0.05). There was no significant difference in serum CA125 levels between the study group and the control group before and after treatment (*P*>0.05). Note: ^*^ compared with before treatment, *P*<0.05. B: The comparison of levels of serum CEA after treatment between the study group and the control group. The serum CEA levels of the study group and the control group after treatment decreased significantly compared with those before treatment (*P*<0.05). There was no significant difference in serum CEA levels between the study group and the control group before and after treatment (*P*>0.05). Note: ^*^ compared with before treatment, *P*<0.05. C: The comparison of levels of serum SCC after treatment between the study group and the control group. The serum SCC levels of the study group and the control group after treatment decreased significantly compared with those before treatment (*P*<0.05). There was no significant difference in serum SCC levels between the study group and the control group before and after treatment (*P*>0.05). Note: ^*^ compared with before treatment, *P*<0.05

The serum CEA, CA125 and SCC levels of the study group and the control group after treatment decreased significantly compared with those before treatment, with statistically significant differences (*P*<0.05). There was no significant difference in serum CEA, CA125 and SCC levels between the study group and the control group before and after treatment (Table 5 and Fig. 2).

**Table 5:** Comparison of tumor markers between the study group and the control group before and after treatment (x±sd)

***Groups***	***CEA(μg/L)***		***CA125(μg/L)***		***SCC(μg/L)***	
	***Before treatment***	***After treatment***	***Before treatment***	***After treatment***	***Before treatment***	***After treatment***
Research group (n=63)	101.33±8.74	3.21±0.58	59.31±4.56	37.46±3.94	6.51±1.58	0.99±0.32
Control group (n=58)	100.59±9.24	2.99±1.11	58.79±5.21	38.13±4.73	6.84±1.24	1.06±0.21
*t*	0.453	1.382	0.585	0.849	1.271	1.410
*P*	0.652	0.170	0.559	0.398	0.206	0.161

## Discussion

Advanced NSCLC is a clinically common malignant tumor disease. At present, advanced NSCLC is the tumor disease with the high morbidity and mortality in the world (13). Although chemotherapy can effectively treat advanced NSCLC (14), patients have different tolerances to different chemotherapy methods. Hence, it is of great significance to explore the medication regimen for chemotherapy to improve the quality of life of patients with advanced NSCLC. Tumor markers are substances generated by abnormal malignant tumor cells which can reflect the occurrence and development of tumor, and monitor tumor response to treatment. Changes in the serum level of tumor markers are closely related to the disease of advanced NSCLC (15, 16).

In clinical practice, the traditional treatment scheme is a combination of platinum-based drugs. But some patients will develop drug resistance to this traditional chemotherapy method, thus affecting the best therapeutic effect. Therefore, the chemotherapy scheme needs to be changed (17, 18). Docetaxel is one of the chemotherapy drugs commonly used in the treatment of advanced NSCLC in clinical practice. It has strong anti-tumor activity, but some clinical studies have shown that use of docetaxel alone can cause sensitization and should be utilized in combination with other drugs (19). Paclitaxel appears to be one of the most promising anti-tumor drugs in the last decade and has shown activity in advanced and refractory ovarian carcinoma, breast cancer, lung cancer, and head and neck cancers. While it is prone to neurotoxicity with paclitaxel (20). Icotinib is generally well tolerated, with mild and reversible adverse reactions, showing positive clinical anti-tumor activity in advanced NSCLC patients (21).

We found that the total clinical effective rate of the study group was not significantly different from that of the control group, but the incidence of leukopenia, oral mucosa ulcer and mild numbness in the control group was significantly higher than that of the study group, indicating that docetaxel combined with icotinib was a feasible treatment for advanced NSCLC with fewer adverse reactions. Although there was no clear clinical comparison, studies have revealed that icotinib-based treatment regimens have a milder response rate and more acceptable toxicity in NSCLC patients (22). CEA is a marker commonly used to detect tumors, which is almost absent in normal tissues (23). CA125 is identified from tumor cell lines (strains) by monoclonal antibody technology, and has high accuracy in the diagnosis of advanced NSCLC (24). SCC is a biomarker that can be used as a prognostic marker for NSCLC patients, and its elevated level may be associated with poor prognosis (25). These three indicators would be at a high level during the period of illness (26). In our study, changes of markers all decreased significantly after chemotherapy in the two group, and the reduction effect of tumor markers after treatment in the study group was slightly better than that in the control group. Our further research found that the ECOG score after chemotherapy was lower than the ECOG score before treatment. ECOG score is an important indicator for predicting the quality of life after treatment. Many related studies have used ECOG scores to understand the improvement of prognosis and quality of life of patients (27). Studies have also shown that icotinib, as an EGFR kinase inhibitor, can prolong progression-free survival and improve quality of life of advanced NSCLC patients (28). This indicates that docetaxel combined with icotinib can improve the quality of life of patients and achieve a desired effect.

Although this study conducted a detailed analysis of docetaxel combined with icotinib in the treatment of advanced NSCLC, there were still deficiencies in the study. We did not utilize docetaxel and icotinib alone. There is a lack of in-depth analysis of the mechanism of action of docetaxel and icotinib on advanced NSCLC. Therefore, there are certain defects in this study, and we will improve the study based on these deficiencies in the later stage, so as to provide better evidence for the research conclusion.

## Conclusion

The application of docetaxel combined with icotinib in the chemotherapy of advanced NSCLC patients can effectively decrease the expression levels of CEA, SCC and CA125 in serum of patients, reduce adverse reactions, and better improve the quality of life of patients, which is worthy of clinical promotion.

## Ethical considerations

Ethical issues (Including plagiarism, informed consent, misconduct, data fabrication and/or falsification, double publication and/or submission, redundancy, etc.) have been completely observed by the authors.
